# p62/SQSTM1-KEAP1 complex prevents clearance of ubiquitinated Z alpha-1 antitrypsin and aggravates liver proteotoxicity

**DOI:** 10.1016/j.jbc.2026.113410

**Published:** 2026-08-07

**Authors:** Nunzia Pastore, Sergio Attanasio, Francesco Annunziata, Claudia D’Agostino, Veronica Maffia, Rita Colonna, Teresa Giuliano, Rosa Ferriero, Iolanda Boffa, Donna Palmer, Philip Ng, Rossella De Cegli, Massimo D’Agostino, Florian Rosenberger, Pavel Strnad, Joseph E. Chambers, Stefan J. Marciniak, Jeffrey Teckman, Pasquale Piccolo, Nicola Brunetti-Pierri

**Affiliations:** 1 Telethon Institute of Genetics and Medicine (TIGEM), Pozzuoli, Naples, Italy; 2 Department of Translational Medicine, Medical Genetics, Federico II University of Naples, Naples, Italy; 3 Department of Molecular and Human Genetics, Baylor College of Medicine, Houston, Texas, USA; 4 Department of Molecular Medicine and Medical Biotechnology, University of Naples Federico II, Naples, Italy; 5 Department of Medical Biochemistry and Biophysics, Division of Molecular Metabolism, Karolinska Institutet, Solna, Sweden; 6 Medical Clinic III, Gastroenterology, Metabolic Diseases, and Intensive Care, University Hospital RWTH Aachen, Aachen, Germany; 7 Cambridge Institute for Medical Research, Keith Peters Building, Cambridge Biomedical Campus, University of Cambridge, Cambridge, UK; 8 Department of Medicine, University of Cambridge, Cambridge, UK; 9 Department of Pediatrics and Department of Biochemistry and Molecular Biology, St Louis University School of Medicine, St Louis, Missouri, USA; 10 Scuola Superiore Meridionale (SSM, School of Advanced Studies), Genomics and Experimental Medicine Program, University of Naples Federico II, Naples, Italy

**Keywords:** AATD, p62/SQSTM1, proteostasis, KEAP1, NRF2

## Abstract

Liver disease in Alpha-1 antitrypsin deficiency (AATD) is caused by the toxic accumulation of mutant Z alpha-1 antitrypsin (Z-AAT) within the endoplasmic reticulum (ER) of hepatocytes. Livers from PiZ transgenic mice expressing the human Z-AAT and AATD patients who are homozygotes for the allele expressing Z-AAT were found to have increased p62/SQSTM1, a multifunctional protein involved in protein homeostasis. The goal of this study was to elucidate the involvement of p62/SQSTM1 in the formation of Z-AAT globules that are responsible for liver injury in AATD. In the present study, we found that p62/SQSTM1 decorated ubiquitin-positive, Periodic-Acid Shiff-diastase-resistant Z-AAT globules and interacted with Z-AAT at the ER-cytosol interface. Genetic ablation of p62/SQSTM1 in PiZ mice (PiZ;*p62*^*−/−*^) led to marked reduction in hepatic Z-AAT globules and polymers, and decreased serum Z-AAT, highlighting a central role for p62/SQSTM1 in disease pathogenesis. Moreover, hepatocyte-specific somatic deletion of the ubiquitin-association (UBA) domain of p62/SQSTM1 reduced Z-AAT aggregation. Furthermore, KEAP1 was identified as a binding partner of p62/SQSTM1-Z-AAT complex, leading to nuclear translocation and activation of NRF2. Inhibition of KEAP1-p62/SQSTM1 interaction reduced the abundance of p62 and phosphorylated p62, decreased intracellular Z-AAT, and redistributed NRF2 to the cytoplasm. In conclusion, this study identifies p62/SQSTM1 as a regulator of Z-AAT proteostasis and link Z-AAT/p62 accumulation to KEAP1 sequestration and NRF2 pathway activation in liver disease due to Z-AAT.

Alpha-1 antitrypsin deficiency (AATD) affects 1 in 2000 to 3000 live births and is caused by pathogenic variants in the *SERPINA1* gene that encodes alpha-1-antitrypsin (AAT) (1). AAT is an acute-phase glycoprotein serine-protease inhibitor (Pi) primarily synthesized by hepatocytes and secreted in plasma. Circulating AAT is delivered to peripheral tissues, most notably the lung, where it acts as a key anti-protease shield by neutralizing neutrophil elastase and related proteases, thereby preserving extracellular matrix integrity and limiting tissue damage (2). Consistent with this role, reduced AAT leads to protease-antiprotease imbalance in the respiratory tract that predisposes to early-onset emphysema.

Among several reported pathogenic *SERPINA1* variants, the Z allele is the most common disease-associated variant. It results from a single amino acid substitution (p.Glu342Lys) that destabilizes the protein structure, resulting in intracellular protein polymerization and reduced blood secretion of AAT (3). In hepatocytes, polymeric Z-AAT accumulates within the endoplasmic reticulum (ER) as Periodic-Acid Shiff-diastase (PAS-D)-positive inclusions, a histopathological hallmark of AATD liver disease. Chronic ER retention triggers proteostasis stress responses and progressive hepatocellular injury, clinically manifesting as chronic hepatitis, fibrosis, cirrhosis, and increased risks of hepatocellular carcinoma (HCC) (4, 5, 6). While AATD-related lung disease results from a loss-of-function due to AAT deficiency, the liver pathology is driven by a gain-of-toxic mechanism associated with intracellular Z-AAT retention.

p62/SQSTM1 (also known as sequestosome-1/SQSTM1) is a multifunctional scaffold protein widely expressed in mammalian tissues (7) that plays central roles in signal transduction, cell proliferation, cell survival, inflammation, tumorigenesis, and oxidative stress response (8). p62/SQSTM1 participates in two major intracellular protein degradation pathways, namely autophagy and the ubiquitin-proteasome system (UPS), both of which have been implicated in the disposal of misfolded Z-AAT protein (9, 10). Through its light chain 3 (LC3)-interacting region (LIR), p62/SQSTM1 binds to LC3 and facilitates selective autophagic degradation of aggregated proteins. Additionally, its ubiquitin-binding domain (UBA) enables recognition and clearance of polyubiquitinated cargo (11), while the N-terminal Phox-BEM1 domain (PB1) supports proteasomal delivery through interaction with the 26S proteasome complex (12). Cytoplasmic inclusions of p62/SQSTM1 have been observed in hepatocytes of patients with AATD, as well as in other proteinopathies and HCC (13). Whether p62/SQSTM1 merely marks Z-AAT globules or plays an active role in Z-AAT proteostasis remains unclear. In this study, we investigated the role of p62/SQSTM1 in mediating Z-AAT-induced liver injury in PiZ mice, a well-established mouse model of AATD-liver pathology (14). PiZ mice are transgenic mice that express the human Z-AAT that is retained in the ER, thus recapitulating the pathology hallmark of the human liver disease. The degree of liver damage correlates with the amount of Z-AAT accumulated in the liver (14).

We showed that p62/SQSTM1 interacts with ubiquitinated Z-AAT at the ER-cytosol interface and promotes an age-dependent accumulation. We further identified a p62/SQSTM1-dependent association of KEAP1 with Z-AAT-positive globules, and we linked these findings to activation of NRF2.

## Results

### p62/SQSTM1 accumulates and binds to ubiquitinated Z-AAT in PiZ mouse livers

**Figure 1:**
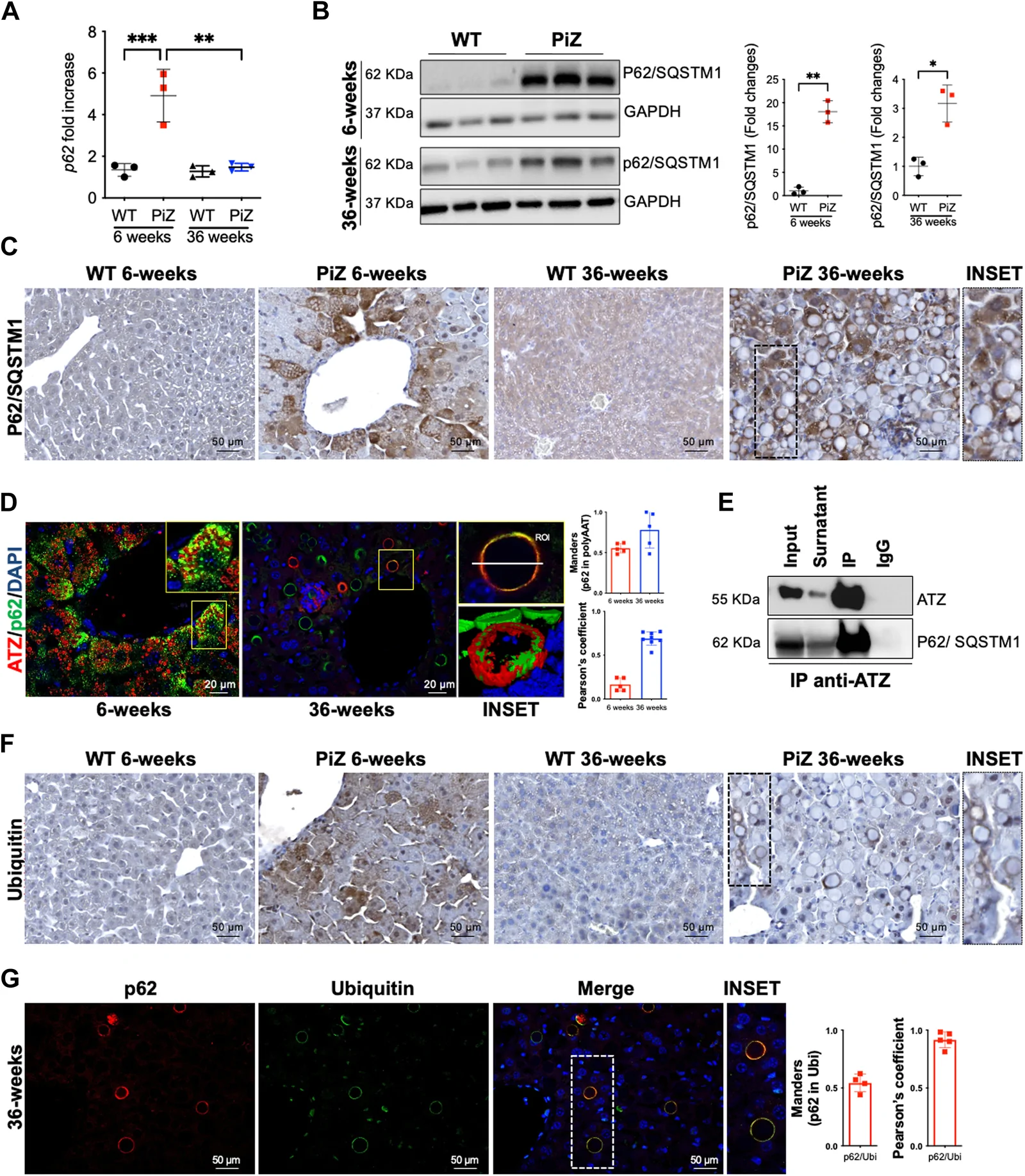
**p62/SQSTM1 is upregulated and co-immunoprecipitates with Z-AAT in PiZ mouse livers.***A*, real-time PCR for *p62/SQSTM1* in wild-type (WT) and PiZ livers at 6- and 36-weeks of age (n = 3 per group). The 6-week-old WT group is set to as 1. *B*, immunoblotting for p62/SQSTM1 in WT and PiZ livers (n = 3 per group) at 6- and 36-weeks of age and corresponding quantifications. GAPDH was used as loading control. Each lane corresponds to an independent mouse. *C*, representative immunohistochemical images of liver sections from WT and PiZ mice stained with anti-p62/SQSTM1 antibody (n = 3 per group). The dotted rectangle indicates the area shown at higher magnification in the inset. *D*, representative immunofluorescences images of livers sections from 6- and 36-week-old PiZ mice stained with anti-p62/SQSTM1 and anti-polymeric Z-AAT antibodies (n = 5 per age group). The *yellow square* indicates the area at higher magnification. The graphs represent the Mander’s colocalization coefficient of p62 in polymeric Z-AAT and the Pearson’s coefficient between *green* and *red* signals. *E*, Immunoblotting for Z-AAT and p62/SQSTM1 on 6-week-old PiZ liver homogenates immunoprecipitated with anti-human AAT antibody. *F*, representative immunohistochemistry images of WT and PiZ livers stained with anti-Ubiquitin antibody. *G*, representative immunofluorescence of 36-week-old PiZ mouse livers co-stained with anti-p62/SQSTM1 and anti-Ubiquitin antibodies. The r*ectangles* outlined by *dashed lines* indicate the area at higher magnification. The graphs represent the Mander’s colocalization coefficient of p62 in ubiquitin and the Pearson’s coefficient between *green* and *red* signals. Averages ± standard deviations are shown. Student’s *t* test: ∗∗*p*-value < 0.01.

*p62/SQSTM1* mRNA expression was approximately 5-fold higher in livers of 6-week-old PiZ mice compared to age-matched wild-type (WT) controls, whereas the upregulation was no longer detected in older 36-week-old PiZ mice (Fig. 1*A*). In contrast, higher p62/SQSTM1 protein amounts were detected by immunoblot and immunohistochemistry in 6- and 36-week-old PiZ livers compared to WT (Fig. 1, *B* and *C*). While its distribution by immunohistochemistry was diffuse in perivenous hepatocytes of 6-week-old PiZ mice, p62/SQSTM1 accumulation was largely located around the larger Z-AAT-positive globules in 36-week-old PiZ mice (Fig. 1*C*). Consistently, p62/SQSTM1 decorated Z-AAT-positive globules, as shown by super-resolution immunofluorescence (Fig. 1*D*) and co-immunoprecipitated with soluble Z-AAT in PiZ mouse livers (Fig. 1*E*). Altogether, these findings are consistent with previously reported interaction between p62/SQSTM1 and Z-AAT in HEK293T cells (15). Consistent with previous findings (16), polyubiquitinated proteins were globally increased in both 6- and 36- week-old PiZ mouse livers compared to age-matched controls (Fig. S1, *A* and *B*) and they showed a diffuse pattern of expression in livers of 6-week-old PiZ mice, whereas a discrete distribution around Z-AAT-positive globules was observed in 36-week-old PiZ mice (Fig. 1*F*). Moreover, p62/SQSTM1 co-localized with ubiquitin around Z-AAT-positive globules by liver immunofluorescence (Fig. 1*G*). Taken together, these data suggest that p62/SQSTM1 accumulates around Z-AAT globules that are polyubiquitinated and binds to Z-AAT in hepatocytes.

**Figure 2:**
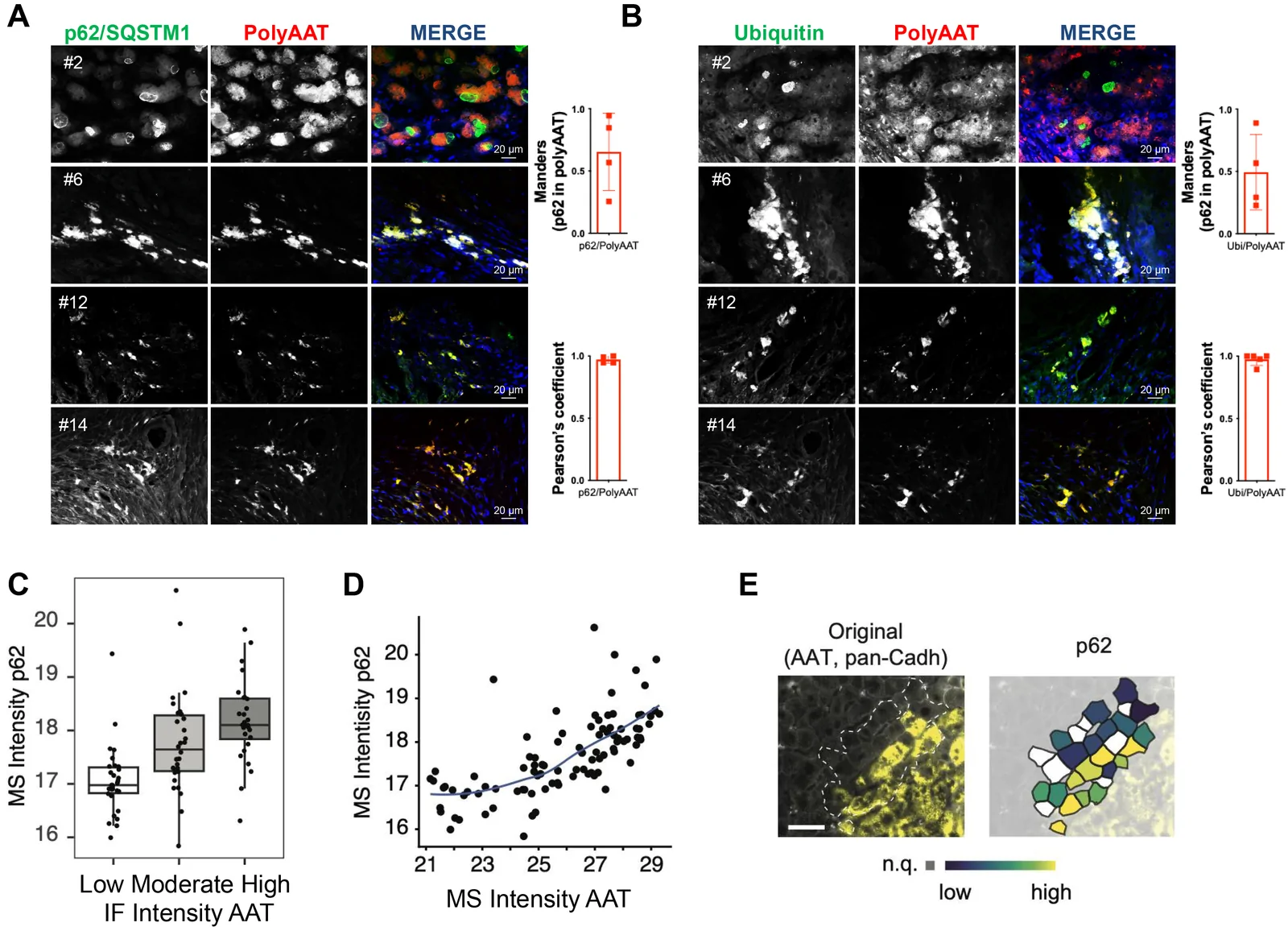
**p62/SQSTM1 and Ubiquitin colocalize with Z-AAT in AATD human livers.***A, B*, Representative immunofluorescence with anti-polymeric Z-AAT and anti-p62/SQSTM1 (*A*) or anti-ubiquitin antibodies (*B*) in livers from four independent patients having AATD with end-stage liver disease and relative quantifications. The graphs represent the Mander’s colocalization coefficient of p62 in polyAAT and the Pearson’s coefficient between *green* and *red* signals. *C*, protein mass spectrometry intensity of p62/SQSTM1 across hepatocyte samples with different visual AAT loads. Each dot represents one sample from a patient (n = 34; 100 laser-microdissected hepatocytes per sample). Boxplots indicate the median (thick line), first and third quartiles (box), and ±1.5 interquartile range (whiskers). *D*, pseudo-time profile of p62/SQSTM1 abundance in spatial proteomics data (n = 34 patients; up to three samples per patient; 100 hepatocytes per sample). A *blueline* indicates a fitted smoothing curve. *E*, Single-hepatocyte quantification of p62/SQSTM1 with the corresponding microscopy image prior to microdissection. The AAT panel, used as a visual control, is the same as reported in Rosenberger *et al.*, 2025 (18). n.q., not quantified. Averages ± standard deviations are shown.

These findings are paralleled in diseased livers of Pi∗ZZ patients (17) that showed p62/SQSTM1 and ubiquitin colocalization with Z-AAT by immunofluorescence, particularly around larger globules (Fig. 2, *A* and *B*). Furthermore, analysis of single-cell spatial proteomics in livers of Pi∗ZZ patients (18) confirmed increased p62/SQSTM1 that closely follows Z-AAT aggregation in hepatocytes (Fig. 2, *C* and *D*). Human hepatocytes devoid of aggregates show no increase of p62/SQSTM1 (Fig. 2*E*), providing independent evidence at the single-cell level that p62/SQSTM1 is engaged in Z-AAT overload.

### Deletion of *p62/SQSTM1* reduces hepatic Z-AAT in adult but not in juvenile PiZ mouse livers

**Figure 3:**
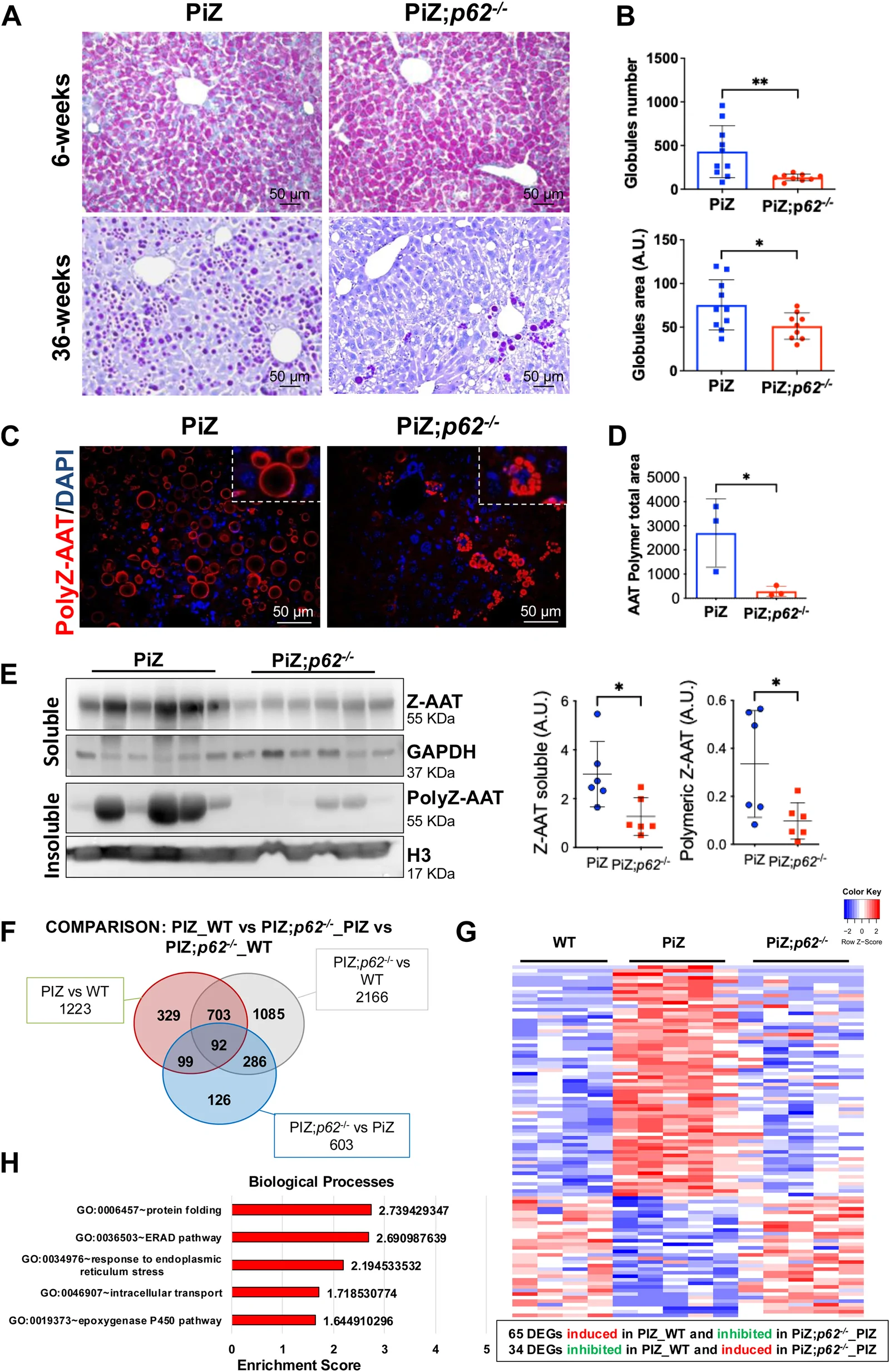
**Deletion of *p62/SQSTM1* reduces hepatic Z-AAT accumulation in 36-week-old PiZ mice.***A*, Representative PAS-D staining of PiZ and PiZ;*p62*^−/−^ mouse livers at 6- and 36-weeks of age. *B*, Quantification of the mean area and number of Z-AAT globules in 36-week-old PiZ and PiZ;*p62*^*−/−*^ mouse livers visualized by PAS-D staining (at least n = 9 per group, dots correspond to the average of n = 5 fields for each mouse). *C and D*, Representative immunofluorescence for polymeric Z-AAT in 36-week-old PiZ and PiZ;*p62*^*−/−*^ mouse livers and relative quantifications. *E*, immunoblotting for soluble and insoluble Z-AAT in 36-week-old PiZ and PiZ;*p62*^*−/−*^ mouse livers. GAPDH and H3 were used as loading controls. *F*, VENN diagram showing overlapping genes among the indicated comparisons. *G*, heatmap displaying the 99 oppositely regulated genes between PiZ and PiZ;*p62*^*−/−*^ mice. *H*, gene ontology (GO) enrichment analysis of the 99 inversely regulated genes shown in (*G*), limited to the Biological Processes category. Each lane corresponds to an independent mouse. Averages ± standard deviations are shown. Student’s *t* test: ∗*p*-value < 0.05.

To investigate the role of p62/SQSTM1 in accumulation of Z-AAT, we crossed PiZ mice with *p62*^*−/−*^ mice (19). While no differences in Z-AAT globules were detected in livers of 6-week-old PiZ;*p62*^*−/−*^ mice, a 70% reduction of PAS-D-positive globule number and 32% of PAS-D area were detected in 36-week-old PiZ;*p62*^*−/−*^ mice (Fig. 3, *A* and *B*). This reduction was accompanied by decreased hepatic Z-AAT polymers detected by immunofluorescence with an anti-polymer antibody in livers of 36-week-old PiZ;*p62*^*−/−*^ mice compared to age-matched PiZ controls (Fig. 3, *C* and *D*). Moreover, serum AAT concentrations were reduced in 36-week-old PiZ;*p62*^*−/−*^ mice compared to PiZ controls (Fig. S2). Furthermore, Z-AAT was reduced in both soluble and insoluble liver fractions in PiZ;*p62*^*−/−*^ mice compared to PiZ controls (Fig. 3*E*). Although fewer PAS-positive globules were still present in older PiZ;*p62*^*−/−*^ mice, these data suggest that p62/SQSTM1 promotes Z-AAT accumulation in an age-dependent manner.

By transcriptome analysis, we investigated the consequences of *p62/SQSTM1* deletion on gene expression profiles of PiZ mice (GSE182961) and the following comparisons were made to isolate genes differentially expressed in PiZ compared to wild-type and dependent on p62/SQSTM1: PiZ *vs.* wild-type, PiZ;*p62*^*−/−*^ *vs.* wild-type, and PiZ;p62^*−/−*^ *vs.* PiZ at 36 weeks of age (Fig. 3*F*). This analysis revealed 99 genes differentially expressed in opposite correlation in PiZ *vs.* wild-type compared to PiZ;*p62*^*−/−*^ *vs.* PiZ (Table S1 and Fig. 3*G*). Gene ontology (GO) analysis showed that genes involved in ER stress response and protein folding were up-regulated in PiZ *vs.* WT and were normalized in PiZ;*p62*^*−/−*^ mice (Table S2 and Fig. 3*H*). Moreover, biological clusters of metabolic processes, including urea cycle were upregulated in PiZ;*p62*^*−/−*^ compared to PiZ mice (Table S2), suggesting an improvement of the metabolic defect previously observed in PiZ mouse livers (20). Altogether, these data showed that genetic ablation of *p62/SQSTM1* reduced hepatic Z-AAT accumulation and corrected changes in gene expression, mainly including ER stress response and metabolic pathway in 36-week-old PiZ mouse livers.

### Hepatocyte-specific post-natal deletion of the UBA domain of p62/SQSTM1 ameliorates liver disease in PiZ mice

**Figure 4:**
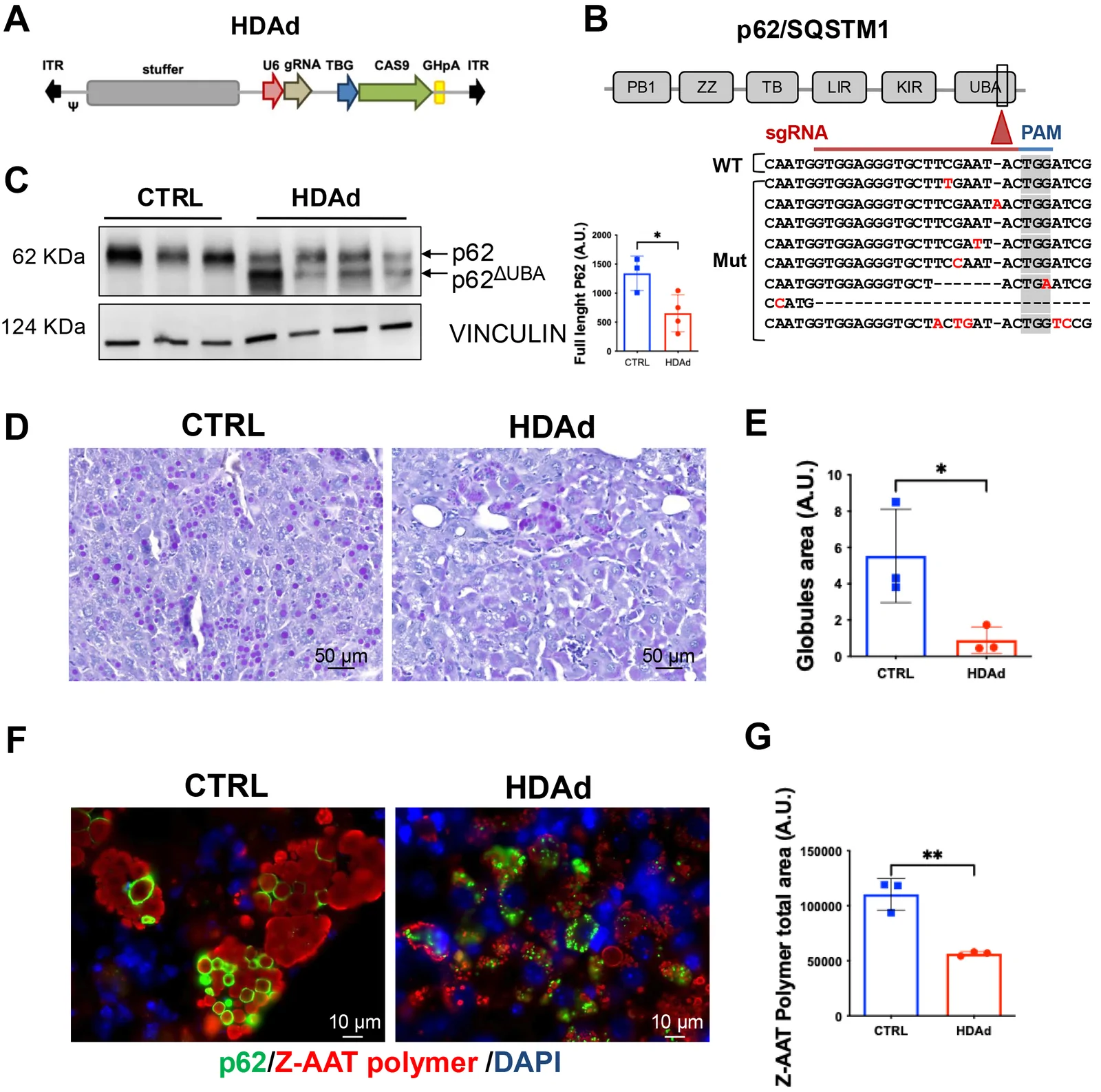
**Somaticdeletion of the UBA domain of *p62/SQSTM1* reduces hepatic Z-AAT accumulation in PiZ mice.***A*, HDAd vector bearing the Cas9 under the control of the hepatocyte-specific TBG promoter and a gRNA targeting *p62/SQSTM1. B*, Representative sequences of indels generated in the *p62/SQSTM1* gene upon gene targeting. *C*, Immunoblotting for p62/SQSTM1 in control mice and in mice injected with the HDAd vector showing the truncated p62/SQSTM1 and relative quantification. Vinculin was used as loading control. *D*, Representative PAS-D staining of control and HDAd injected PiZ mouse livers harvested 12 weeks after HDAd injections. *E*, quantification of the mean area of the Z-AAT globules visualized by PAS-D staining in livers of HDAd-injected PiZ mice (at least n = 3 per group, dots correspond to the average of n = 10 fields for each mouse). Representative immunofluorescence (*F*) and relative quantification (*G*) of polymeric Z-AAT and p62/SQSTM1 in livers of HDAd-injected and control PiZ mice. Averages ± standard deviations are shown. Student’s *t* test: ∗*p*-value < 0.05; ∗∗*p*-value < 0.01.

The UBA domain of p62/SQSTM1 binds poly-ubiquitin chains of proteins and this UBA domain is hypothesized to be involved in the binding of p62/SQSTM1 to Z-AAT (15). To confirm this hypothesis, we injected PiZ mice with the hepatotropic helper-dependent adenoviral (HDAd) vector (21) expressing Cas9 under the control of the liver-specific promoter thyroxine-binding globulin (TBG) and a guide RNA (gRNA) under the control of the U6 promoter (HDAd-Cas9-UBA-gRNA) to delete the UBA domain of p62/SQSTM1 in hepatocytes (Fig. 4*A*). DNA sequencing and immunoblotting on livers harvested post-injection confirmed that the Cas9 and gRNA delivered by the HDAd showed indels in the murine *p62/SQSTM1* gene (Fig. 4*B*) that resulted in truncated forms of p62/SQSMT1^ΔUBA^ and reduced full-length p62/SQSTM1 protein (Fig. 4*C*). Livers of HDAd-injected PiZ mice showed reduced Z-AAT by PAS-D staining (Fig. 4, *D* and *E*) and compared to control mice, HDAd-injected PiZ mouse livers showed smaller globules not decorated by p62/SQSTM1 (Fig. 4, *F* and *G*). Therefore, either complete abrogation of p62/SQSTM1 or selective deletion of the UBA domain of p62/SQSTM1 reduces Z-AAT globules, suggesting that the UBA domain of p62/SQSTM1 promotes Z-AAT aggregation.

### Z-AAT and p62/SQSTM1 interact at the ER-cytosol interface

**Figure 5:**
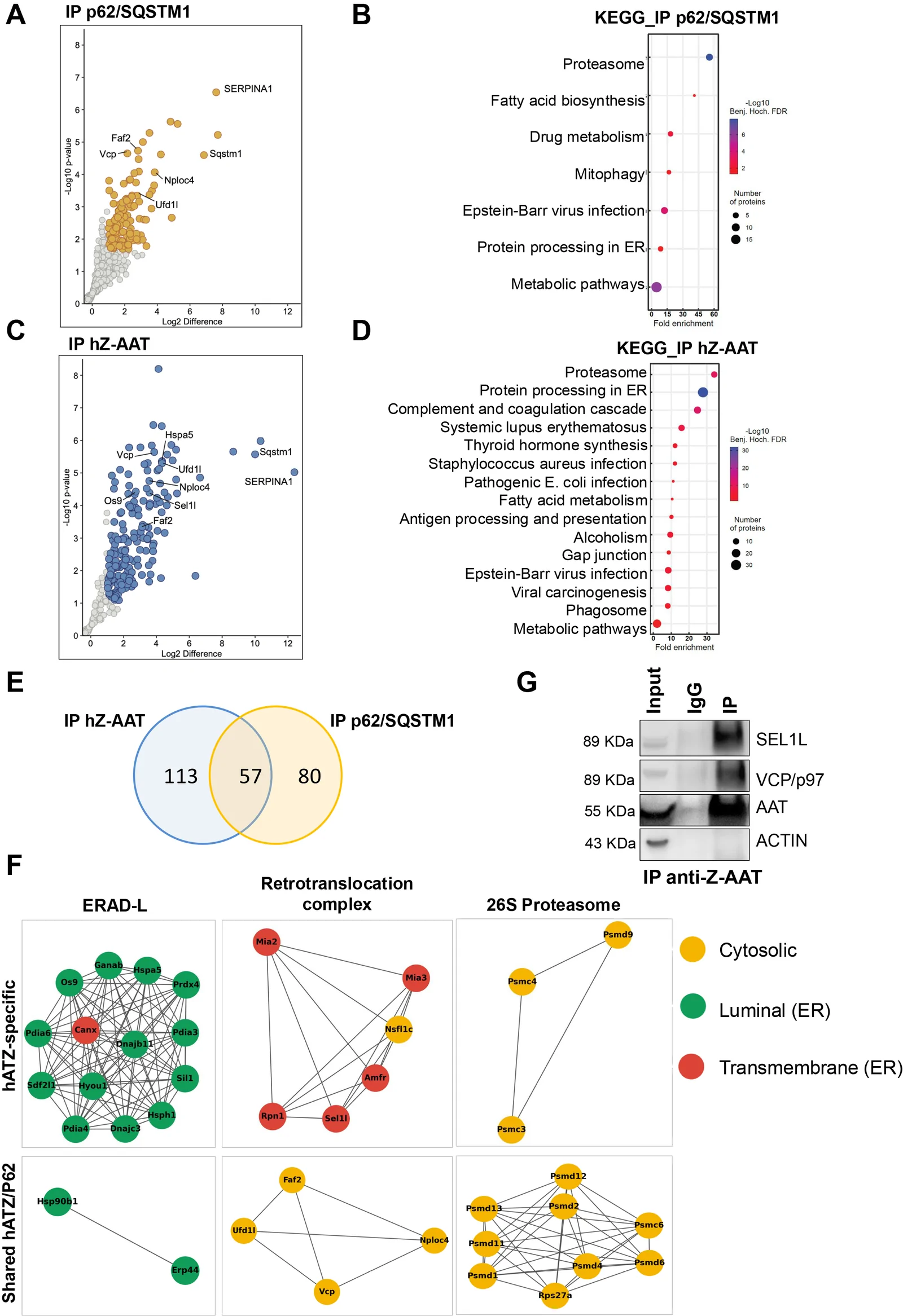
**ERAD and proteasomal proteins co-immunoprecipitate with Z-AAT and p62/SQSTM1.***A*, Volcano plot of mass spectrometry (MS) analysis of p62/SQSTM1 interactors in 6-week-old PiZ mouse livers (n = 4 per group). *B*, KEGG pathways enriched in the co-IP studies with p62/SQSTM1 in 6-week-old PiZ mouse livers. *C*, Volcano plot of MS analysis of Z-AAT interactors in 6-week-old PiZ mouse livers (n = 4 per group). *D*, KEGG pathways enriched in the co-IP studies with soluble Z-AAT in 6-week-old PiZ mouse livers. Venn diagram (*E*) and network analysis (*F*) of the proteins significantly enriched in the two IP experiments. Proteins enriched exclusively in the Z-AAT IP or shared between Z-AAT and p62/SQSTM1 IPs were assigned to ERAD-L, retrotranslocation/extraction, or 26S proteasome modules. The p62/SQSTM1-only interactome did not contain ERAD components. *G*, immunoblotting for selected proteins on immunoprecipitated 6-week-old PiZ liver homogenates with anti-human AAT antibody.

To understand how p62/SQSTM1 promotes Z-AAT aggregation, we performed an unbiased proteomic analysis of p62/SQSTM1 and Z-AAT interactomes on livers from 6-week-old PiZ mice. The analysis identified 137 proteins significantly enriched by immunoprecipitation (IP) with the anti-p62/SQSTM1 antibody and 170 proteins significantly enriched with the anti-total Z-AAT antibody (Fig. 5, *A* and *C* and Table S3). Gene Ontology (GO) analysis revealed that most of these interacting proteins were associated with proteasomal function and ER-associated degradation (ERAD) pathways (Fig. 5, *B* and *D* and Table S4). Notably, 57 proteins were co-immunoprecipitated with both p62/SQSTM1 and Z-AAT antibodies (Fig. 5*E*), including canonical ER-luminal (ERAD-L) proteins (VCP, UFD1, FAF2, and Nploc4) and proteins of the 26S proteasome. Topology-based network analysis showed that proteins uniquely immunoprecipitated with Z-AAT were mainly ER-luminal and transmembrane factors, whereas proteins shared between Z-AAT and p62/SQSTM1 were largely cytosolic and belonged to the proteasomal degradation (Fig. 5*F*). Z-AAT-specific interactome was enriched for ER-resident luminal chaperones and transmembrane ERAD adaptors, including OS9, BiP/HSPA5, ERP44, and SEL1L, suggesting engagement of proteins of the ERAD-L quality control and luminal recognition steps. The interaction of representative ERAD components with Z-AAT, including SEL1L and VCP/p97, was validated by co-immunoprecipitation followed by immunoblotting (Fig. 5*G*). Altogether, these data suggest that Z-AAT and p62/SQSTM1 physically converge at the ER-cytosol interface, where Z-AAT interacted primarily with luminal and membrane-anchored ERAD components, whereas p62/SQSTM1 associated with cytosolic extraction and proteasomal complexes.

### p62/SQSTM1-dependent recruitment of KEAP1 to Z-AAT-positive globules is associated with activation of NRF2

**Figure 6:**
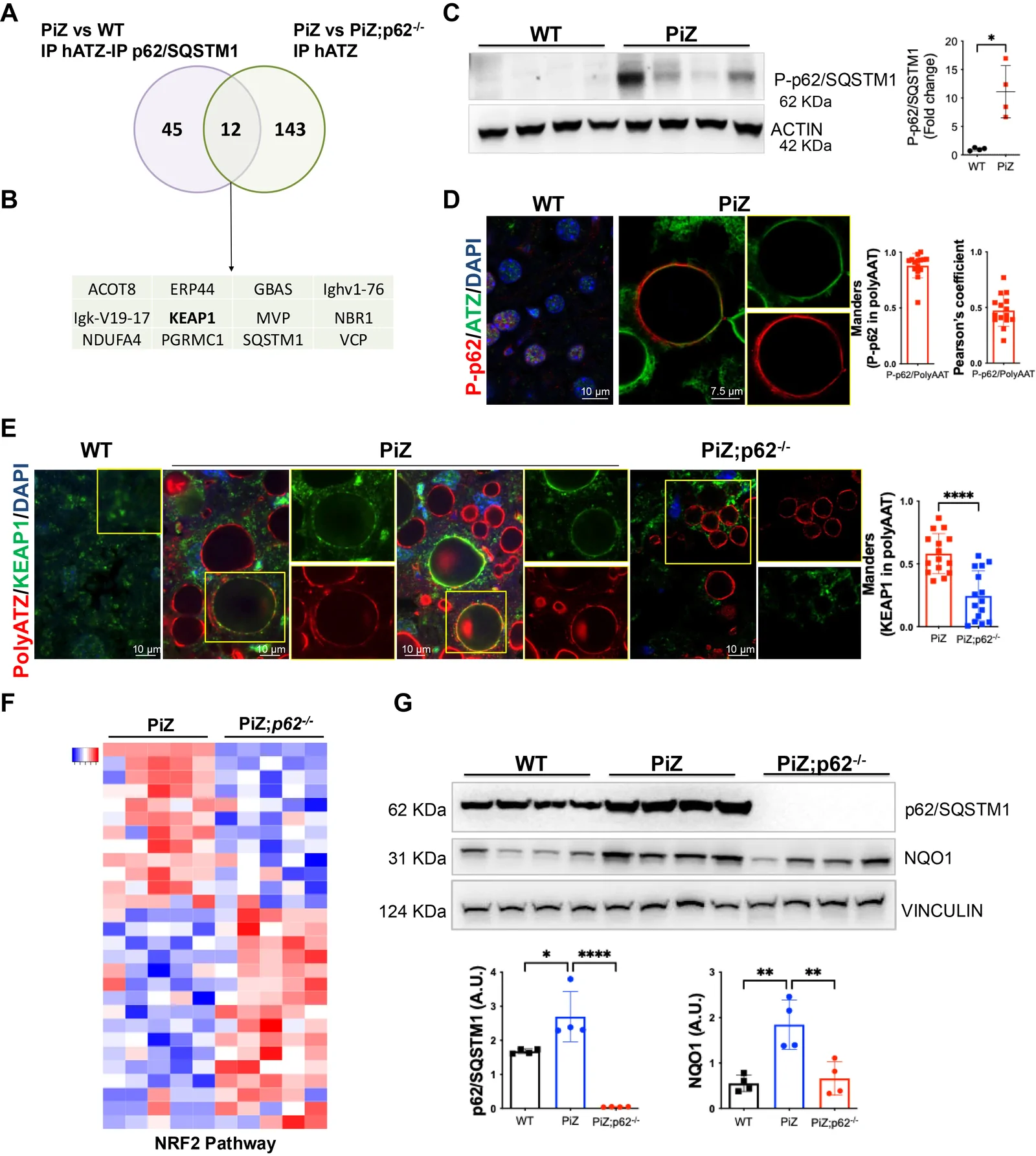
**p62/SQSTM1 interacts with KEAP1 and activated NRF2 pathway.** (*A*) VENN diagram of the common proteins immunoprecipitated with Z-AAT and p62/SQSTM1 antibody in PiZ livers and those enriched in PiZ *vs* PiZ;*p62*^−/−^ mouse livers at 36 weeks of age. (*B*) List of the 12 common proteins from the VENN diagram reported in (*A*). (*C*, *D*) Immunoblot (*C*) and Immunofluorescence (*D*) analysis for phosphorylated p62 in WT and PiZ livers with relative quantifications. The graphs represent the Mander’s colocalization coefficient of Phospo-p62 in polymeric Z-AAT and the Pearson’s coefficient between *green* and *red* signals. (*E*) Immunofluorescence analysis for KEAP1 and polymeric Z-AAT in liver sections from 36-week-old WT, PiZ and PiZ;*p62*^*−/−*^ mice with relative quantifications. The graphs represent the Mander’s colocalization coefficient of KEAP1 in polyAAT and the Pearson’s coefficient between green and red signals. (*F*) Heatmap displaying the NRF2 pathway-related genes oppositely regulated between PiZ and PiZ;*p62*^*−/−*^ mice. (*G*) Immunoblot analysis for p62/SQSTM1 and NQO1 in liver lysates of 36-week-old WT, PiZ, and PiZ;*p62*^−/−^ mice and relative quantification (n = 4 per group). Vinculin was used as loading control. Averages ± standard deviations are shown. Each dot represents an individual mouse. ANOVA: ∗*p*-value < 0.05; ∗∗∗*p*-value < 0.001.

Comparative proteomics in livers from 36-week-old PiZ and in PiZ;*p62*^*−/−*^ mice revealed 155 proteins significantly enriched in Z-AAT IP from PiZ livers compared to PiZ;*p62*^*−/−*^ (Fig. S3, *A*, *B* and Tables S5 and S6). Twelve proteins (COT8, ERP44, GBAS, Ighv1-76, Igk-V19-17, KEAP1, MVP, NBR1, NDUFA4, PGRMC1, SQSTM1, and VCP) were commonly enriched in both the comparisons between p62/SQSTM1 and Z-AAT IP in PiZ *vs.* WT mice and the protein enriched in PiZ *vs.* PiZ;*p62*^*−/−*^ (Fig. 6, *A* and *B*), suggesting their dependence on p62/SQSTM1. Besides p62/SQSTM1, this group included proteins involved in selective autophagy and ubiquitin-dependent protein quality control, such as NBR1 (22) and VCP (23), ER proteostasis-associated proteins, such as ERP44 (24) and PGRMC1 (25), and proteins involved in cellular metabolism or stress signaling, such as ACOT8 (26), NDUFA4 (27), and MVP (28). KEAP1, a central repressor of the NRF2 pathway, was prioritized given its established binding to phosphorylated p62/SQSTM1 (29). Supporting a KEAP1-binding-competent p62/SQSTM1 (29), phosphorylated p62/SQSTM1 was increased in PiZ compared to WT livers and localized around the Z-AAT-positive globules (Fig. 6, *C* and *D*). Immunofluorescence analysis showed that KEAP1 was enriched around Z-AAT globules in PiZ livers (Fig. 6*E*). In contrast, KEAP1 staining was diffuse, and its co-localization with Z-AAT was markedly reduced in PiZ;*p62*^*−/−*^livers (Fig. 6*E*), suggesting a role for p62/SQSTM1 in the recruitment of KEAP1 to Z-AAT-globules. Based on these findings, we hypothesized that KEAP1 is sequestered by the Z-AAT-p62/SQSTM1 complex, leading to activation of the NRF2 transcriptional program (30). In line with this hypothesis, Gene Set Enrichment Analysis (GSEA) showed robust NRF2 pathway activation in PiZ livers compared to WT, which was significantly blunted in at 36-week-old PiZ;*p62*^*−/−*^ (Figs. 6*F* and S4; Table S7). NRF2 pathway activation was confirmed by immunoblot analysis for NQO1, a canonical NRF2 target (Fig. 6*G*). Taken together, these data suggest that the recruitment of KEAP1 by p62/SQSTM1 binding Z-AAT during its retro-translocation from the ER lumen to the cytosol leads to aberrantly increased activation of NRF2 signaling.

### Inhibition of KEAP1-p62/SQSTM1 interaction promotes Z-AAT clearance

**Figure 7:**
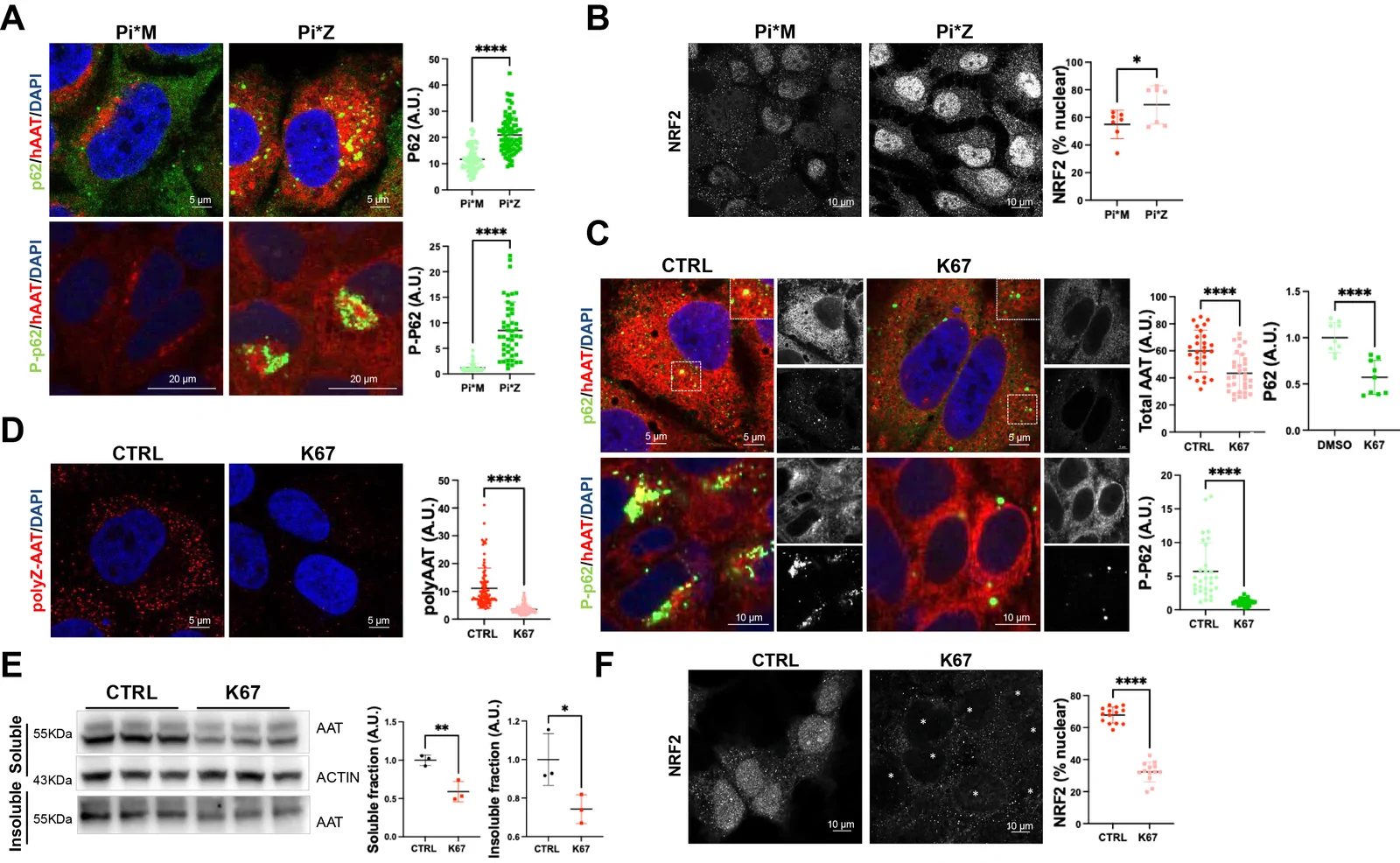
**Inhibition of p62/SQSTM1-KEAP1 binding increases Z-AAT degradation.***A*, Immunofluorescence analysis for p62/SQSTM1 or phospho-p62 and total AAT in Pi∗M and Pi∗Z Huh7.5 cells with relative quantification. *B*, immunofluorescence for NRF2 in Pi∗M and Pi∗Z Huh7.5 cells and relative quantification. *C, D*, immunofluorescence analysis for total AAT and p62/SQSTM1 or phospho-p62 (C) and for polymeric Z-AAT (D) in Pi∗Z Huh7.5 cells treated with K67 or vehicle as control with relative quantifications. *E*, Immunoblot analysis for soluble and insoluble forms of AAT from Pi∗Z Huh7.5 treated with K67 compared to control cells and relative quantifications. *F*, immunofluorescence for NRF2 in Pi∗Z Huh7.5 cells treated with K67 or vehicle and relative quantification. MFI: mean fluorescence intensity. Each dot represents an individual cell. Averages ± standard deviations are shown. Student’s *t* test: ∗*p*-value < 0.05; ∗∗*p*-value < 0.01; ∗∗∗*p*-value < 0.001.

To further investigate the mechanism underlying the detrimental effect of the p62/SQSTM1-KEAP1 on liver disease induced by Z-AAT, we used Pi∗Z Huh7.5 cells, a hepatocyte cell line with the normal AAT (PI∗M) gene edited to express the Z-AAT (31). As controls, Pi∗M Huh7.5 cells expressing wild-type *SERPINA1* (31) were used. In contrast to Pi∗M Huh7.5 cells, Pi∗Z Huh7.5 cells were found to accumulate intracellular Z-AAT polymers and aggregates of p62/SQSTM1 and phosphorylated p62 (Fig. 7*A*). Consistent with the *in vivo* data, Pi∗Z Huh7.5 cells showed increased nuclear NRF2 compared to control cells (Fig. 7*B*). Treatment of Pi∗Z Huh7.5 cells with K67, a selective small-molecule inhibitor of the interaction between KEAP1 and p62/SQSTM1 (32), reduced both total and polymeric Z-AAT (Fig. 7, *C*–*E*). K67 also reduced both total and phosphorylated p62/SQSTM1 (Fig. 7*C*). Moreover, K67 induced relocation of NRF2 to the cytoplasm (Fig. 7*F*). These data suggest that disruption of KEAP1-p62/SQSTM1 interaction affects the positive feedback loop promoting NRF2 activation, thus favoring Z-AAT clearance.

**Figure 8:**
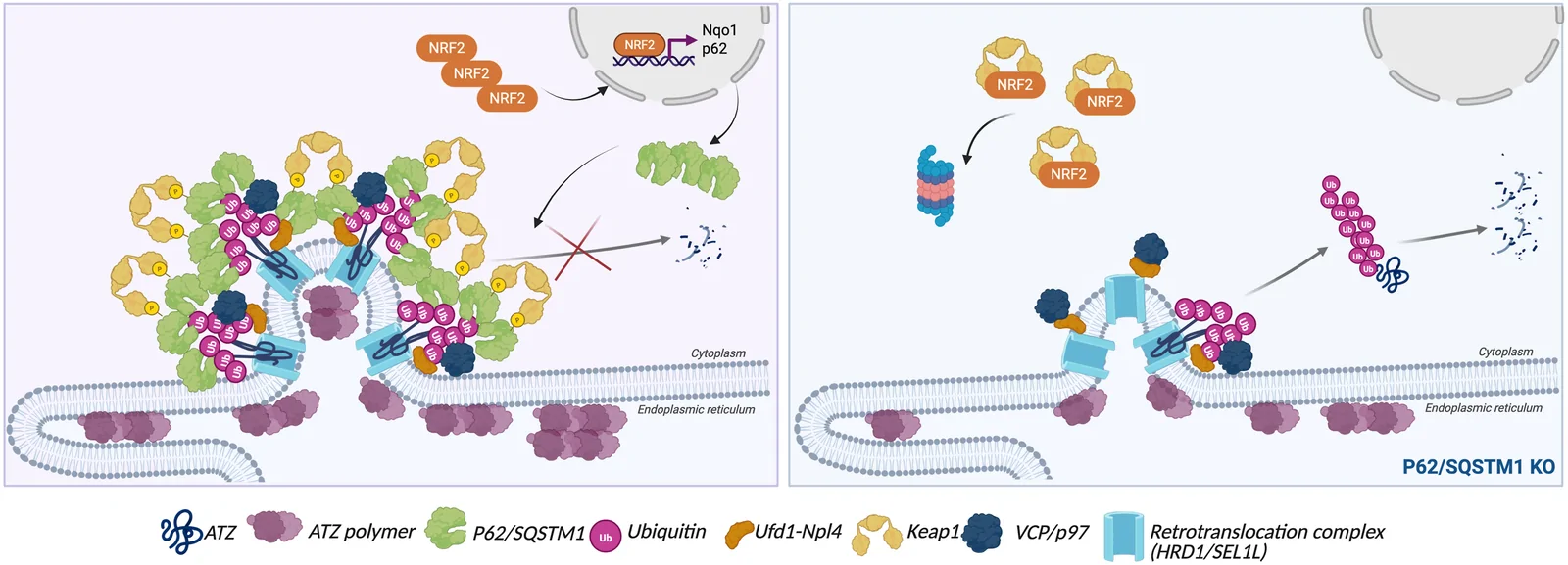
**Proposed model for p62/SQSTM1 impairing clearance of Z-AAT.** P62/SQSTM1 sequesters ubiquitinated Z-AAT at the ER membrane during its retrotranslocation *via* the ERAD system, thereby hindering its clearance, recruits KEAP1, and activates NRF2 pathway.

Collectively, these data support a model in which p62/SQSTM1 accumulates around ubiquitinated Z-AAT at the cytosolic face of the ER where KEAP1 is recruited and sequestered. These findings were also associated with NRF2 stabilization and its transcriptional activation (Fig. 8).

## Discussion

Proteasome and autophagy are both involved in the disposal of soluble and aggregated Z-AAT, respectively (9, 33, 34). The proteasome mediates degradation of Z-AAT through the ERAD system (35), which includes its recognition by ER-resident chaperones, retro-translocation into the cytosol, ubiquitination, and subsequent proteasome-mediated degradation (36, 37). p62/SQSTM1 plays a key role in this process by recognizing polyubiquitinated substrates and facilitating their delivery to the proteasome (38). p62/SQSTM1 is a multifunctional adaptor protein containing several structured domains: a PB1 domain, a ZZ-type zinc finger domain, a nuclear localization signal, an export motif, a LIR, a KEAP1-interacting region (KIR), and a C-terminal UBA (39). Through the UBA domain, p62/SQSTM1 binds non-covalently ubiquitinated substrates and routes them either to the proteasome (*via* PB1) or to autophagosomes (*via* LIR) (40). However, excessive p62/SQSTM1 accumulation can paradoxically delay the clearance of ubiquitinated proteins and impair proteasomal efficiency (41).

The present study investigated the incompletely defined role of p62/SQSTM1 in the pathogenesis of the liver disease induced by Z-AAT. Previous work in cultured cells showed that p62/SQSTM1 is associated with Z-AAT (15). Furthermore, p62-positive inclusions were previously detected in AATD livers (13). Here, we show that p62/SQSTM1 is not a simple marker of Z-AAT inclusions, but it binds to ubiquitinated Z-AAT and ERAD components at the ER-cytosol interface and is involved in aggravating the burden of Z-AAT. Z-AAT globules are directly responsible for liver injury, and thus, the reduction of globules by p62/SQSTM1 genetic ablation or hepatocyte-specific deletion results in reduced hepatocyte damage.

Although HRD1, an E3 ligase previously implicated in Z-AAT ubiquitination (15, 42), was not detected by our proteomic studies, several HRD1-associated ERAD components, including VCP, UFD1, NPLOC4, and SEL1L were identified. Proteins of the Z-AAT interactome were largely luminal or membrane-associated, whereas interacting proteins shared with p62/SQSTM1 were mainly cytosolic components of the proteasomal machinery. This topological separation suggests an interaction between Z-AAT and p62/SQSTM1 occurring during or after Z-AAT retrotranslocation at the cytosolic face of the ER.

Z-AAT polymerization leads to ER fragmentation into large vesicular structures known as ER inclusions, which cause hepatocyte injury leading to liver cirrhosis that accounts for 10% of deaths in individuals homozygous for the Z allele (43, 44). Moreover, Z-AAT polymers alter the biophysical properties of the protein milieu within the ER lumen, forming a solid matrix that fills ER inclusions and impairs the mobility of other ER proteins (45). Although polymer accumulation alone does not trigger the UPR, this ER solidification can eventually impair chaperone function and other ER proteostasis (45). In the present study, p62/SQSTM1 protein remained elevated in adult PiZ liver despite normalization of *Sqstm1* mRNA, and its localization shifted from a diffuse pattern in juvenile mice to peri-globular accumulation in adults. This age-dependent redistribution paralleled the finding of reduced Z-AAT accumulation in adult, but not juvenile PiZ mice deleted for p62/SQSTM1. These observations suggest a detrimental role of p62/SQSTM1 when the Z-AAT burden increases or when the proteostasis capacity declines with aging.

The reduction of Z-AAT accumulation after hepatocyte-specific deletion of the UBA domain supports a direct role for ubiquitin recognition. We propose that increased concentrations of p62/SQSTM1 at the ER membrane that binds retrotranslocating and ubiquinated Z-AAT favor its sequestration within an ER-associated protein-rich compartment. Under conditions of chronic Z-AAT overload, this maladaptive response might interfere with efficient substrate processing and degradation (Fig. 8). The loss of the UBA domain indeed prevented p62/SQSTM1 from stably anchoring to ubiquitinated Z-AAT, thereby reducing the formation of large Z-AAT globules.

Among common interacting proteins of p62/SQSTM1 and Z-AAT, we focused on KEAP1 given the established role of p62/SQSTM1 in regulating the KEAP1-NRF2 signaling pathway, a major oxidative stress response system (46). While under physiologic conditions, KEAP1 promotes NRF2 proteasomal degradation, upon binding of phosphorylated p62/SQSTM1 (47), KEAP1 is known to be sequestered, leading to NRF2 stabilization and nuclear translocation (48). Consistently, we found increased phosphorylated p62/SQSTM1 partially overlapping with Z-AAT-positive globules, and enrichment of KEAP1 in PiZ livers. Furthermore, these changes were accompanied by activation of NRF2-responsive genes and increased in its canonical target NQO1. Because it is an NRF2 target gene (49), p62/SQSTM1 establishes a feed-forward loop that amplifies its own expression and exacerbates the proteostatic defect.

In summary, our data support a model by which excessive p62/SQSTM1 in complex with KEAP1 impairs Z-AAT degradation through sequestration of ubiquitinated Z-AAT at the ER-cytosol interface that promotes the formation of large Z-AAT-containing aggregates.

Our data in a cell model suggest that KEAP1 interaction with p62/SQSTM1 prevents Z-AAT degradation, but the involved degradative pathway has to be determined.

Deletion of p62/SQSTM1 reduced the number and size of Z-AAT globules only in adult PiZ mice, supporting distinct mechanisms in juvenile *versus* adult liver disease (50). Liver disease in AATD predisposes to HCC (51), and p62/SQSTM1 is increasingly recognized as a driver of tumorigenesis, particularly through persistent NRF2 activation (8). Mallory-Denk bodies, which are positive for p62/SQSTM1, KEAP1, and ubiquitin are commonly seen in Metabolic Dysfunction-Associated Steatotic Liver Disease (MASLD) and HCC (30, 52). Thus, targeting KEAP1-p62/SQSTM1 interactions may represent a therapeutic strategy for a broad group of liver diseases.

In conclusion, our study identifies p62/SQSTM1 as a critical mediator of liver injury in AATD. By binding Z-AAT and KEAP1 at the ER membrane, p62/SQSTM1 impairs Z-AAT degradation and activates NRF2 signaling, contributing to hepatocellular damage. Genetically or pharmacologically disruption of this complex restores proteostasis and reduces liver injury, offering novel therapeutic opportunities for AATD-related liver disease.

## Experimental procedures

### Mouse experiments

Animal care and procedures were approved by the Italian Ministry of Health. PiZ transgenic mice (14) were maintained on a C57BL/6J background, and only male mice were used. *p62*^*−/−*^ (19) mice were kindly provided by Simone Cenci, San Raffaele University Hospital, Milan, Italy and crossed with the PiZ mice. Genotyping for the *p62/SQSTM1* allele was performed by PCR on genomic DNA extracted from mouse tails using primers listed in Table S8. Blood samples were collected by retro-orbital bleedings, and sera were analyzed for Z-AAT by ELISA as previously described (17, 53). Intravenous injections of HDAd vector were performed by retro-orbital injections of 200 μl at the dose of 1 × 10^13^ viral particles per kg of body weight. Age-matched mice were injected with saline as a control.

### HDAd cloning and production

Single guide RNA (sgRNA) for *p62/SQSTM1* was designed using the CRISPR/Cas9 Target online predictor (CCTop) tool (54). Oligonucleotides (Table S9) for *sgSqstm1* were annealed and cloned into *Bsm*BI sites of lenti-TBG-Cas9-U6 vector, that included the liver-specific thyroxine-binding globulin (TBG) promoter driving the expression of Cas9. The DNA sequence for the expression cassette (TBG-Cas9-U6-sgSqstm1) was excised using *Not*I and *Sma*I and cloned into the *Swa*I site of the HDAd vector plasmid to generate the HDAd-TBG-Cas9-U6-sgSqstm1 vector. HDAd were produced in 116 cells with the helper virus AdNG163 as described in detail elsewhere (55, 56).

### Liver stainings

PAS-D staining was performed on 10-μm-thick paraffin sections of livers. Liver specimens were fixed in 4% PFA for 12 h and stored in 70% Ethanol and embedded into paraffin blocks. The sections were rehydrated and treated with amylase solution 0.5% (α-amylase type VI-B Sigma-Aldrich) for 20 min and then stained with PAS-D reagent according to the manufacturer’s instructions (Bio-Optica, Milan, Italy). For immunostaining, 5-μm sections were rehydrated, hydrated in PBS pH 7.4 and permeabilized with PBS, 0.2% Triton X-100. Heat Induced Epitope Retrieval using citrate buffer method (pH 6.0) was performed to retrieve the antigen sites. Sections were then incubated for 1 h at room temperature with blocking solution (3% BSA (Sigma), 5% donkey serum (Millipore), 1.5% horse serum (Vector Laboratories), 20 mM MgCl2, 0.3% Triton X-100 (Sigma) in PBS pH 7.4) and overnight at 4 °C with primary antibodies (Table S10). Secondary antibodies made in donkey were AlexaFluor-594 anti-rabbit (Invitrogen) and AlexaFluor-488 anti-mouse (Invitrogen). Nuclei were counterstained with DAPI (Invitrogen). For immunohistochemistry, sections underwent blocking of endogenous peroxidase activity in methanol plus 1.5% H_2_O_2_ (Sigma) for 30 min and then, they were incubated with blocking solution. Vectastain ABC kit and DAB (Vector Laboratories) were used according to the manufacturer’s instructions. Mayer’s hematoxylin (Bio-Optica) was used for counterstaining. Sections were dehydrated and mounted in Vectashield (Vector Laboratories). Stained liver sections were examined under a Zeiss LSM700 for fluorescence and Leica DM5000 microscope for brightfield. Quantification of the percentage area and number of Z-AAT globules were performed using the ImageJ plug-in “Analyze particles” on the segmented images. Five different randomly selected fields of PiZ and PiZ;*p62*^*−/−*^ mice (n = 3 per group) were used for quantification.

For immunofluorescence duplex analyses for polymeric Z-AAT/KEAP1 and Z-AAT/p-p62, slides were incubated with antibodies titered with a blocking solution into user-fillable dispensers for use on the automated stainer and then developed using the Discovery FAM kit (cat #760–243) and Discovery RED 610 kit (cat #760–245) according to the manufacturer’s instructions. Nuclei were stained with DAPI (Thermo Fisher Scientific, D1306). Stained liver sections were examined under a Zeiss Axiocam MR microscope or with ZEISS Axio Scan.Z1.

### Colocalization analysis

Fluorescence images were analyzed by restricting the measurements to automatically segmented circular red-positive regions of interest (ROIs). Red-specific structures were identified from a red-enhanced image, thresholded using Otsu’s method, and filtered by size and shape criteria. Pearson’s correlation coefficient was calculated from paired red and green pixel intensities within each ROI. Thresholded Manders analysis was calculated as the fraction of green fluorescence intensity located in pixels positive for both red and green within the same ROI.

### Real-time PCR

Total RNA was extracted from liver tissue in TRIzol reagent (Invitrogen) using RNeasy kit (Qiagen). RNA was reverse transcribed using a first strand complementary deoxyribonucleic acid kit with random primers according to the manufacturer’s protocol (Applied Biosystems). RT-PCR reactions were performed by the Roche Light Cycler 480 system (Roche). The PCR reaction was performed using the SYBR Green Master Mix (Roche). PCR conditions for all tested genes were as follows: pre-heating, 5 min at 95 °C; cycling, 40 cycles of 15 s at 95 °C, 15 s at 60 °C and 25 s at 72 °C. Results were expressed in terms of cycle threshold (C_t_). The C_t_ values were averaged for each duplicate. ß2-microglobulin was used as an endogenous control gene (reference marker). Data were analyzed using LighCycler 480 software, version 1.5 (Roche Applied Science). Primers are listed in Table S11.

### Immunoblotting

Liver samples were homogenized in RIPA buffer (50 mM Tris–HCl pH 7.4, 150 mM NaCl, 1% Triton X-100, 1 mM EDTA pH 8.0, 0.1% SDS) containing complete protease inhibitor cocktail and phosphatase inhibitor (Roche Applied Science). Samples were incubated for 20 min at 4 °C and centrifuged at 16,000*g* for 10 min. The pellet was discarded, and cell lysates were used for immunoblots. 10 to 20 μg of liver lysate was electrophoresed on a 12% SDS-PAGE. After transfer to a nitrocellulose membrane, blots were blocked in TBS-Tween20 containing 5% non-fat milk for 1 h at room temperature, and the primary antibody was next applied overnight at 4 °C. Anti-rabbit IgG, anti-mouse IgG, or anti-goat-IgG conjugated with horseradish peroxidase (GE Healthcare, 1:3000) and ECL (Pierce) were used for detection. Equal gel loading was confirmed with immunoblot for GAPDH (Santa Cruz) or β-Actin (Novus Biological). Primary antibodies are listed in Table S3. Preparation of soluble and insoluble fractions from livers was performed as previously described (50), and immunoblotting was performed using antibodies against the total AAT and the polymeric Z-AAT.

### QuantSeq 3′ mRNA sequencing

RNA was extracted from livers of wild-type (n = 4), PiZ (n = 5), and PiZ;p62^−/−^ (n = 5) mice aged 36 weeks. Total RNA was precisely quantified by Qubit 2.0 fluorimetric Assay (Thermo Fisher Scientific). Preparation of libraries was performed with a total of 125 ng of RNA from each sample, using the QuantSeq 3′mRNA-Seq Library prep kit FWD for Illumina (Lexogen GmbH) according to the manufacturer’s instructions. Quality of the libraries was assessed by using ScreenTape High sensitivity DNA D1000 (Agilent Technologies). The amplified fragmented cDNA of 300 bp in size was sequenced in single-end mode using the Illumina NovaSeq 6000 with a read length of 100 bp. Illumina NovaSeq base call (BCL) files were converted into fastq file through bcl2fastq (https://support.illumina.com/downloads/bcl2fastq2-conversion-user-guide-v2.html) (version 2.20.0.422). For analysis, sequence reads were trimmed using bbduk software (https://jgi.doe.gov/data-and-tools/bbtools/bb-tools-user-guide/usage-guide/) (bbmap suite 37.31) to remove adapter sequences, poly(A) tails, and low-quality end bases (regions with average quality below 6). Alignment was performed with STAR 2.6.0a (57) on the mm10 reference assembly obtained from cellRanger website (https://support.10xgenomics.com/single-cell-gene-expression/software/release-notes/build#mm10_3.0.0; Ensembl assembly release 93). Gene expressions were determined with htseq-count (58) using the Gencode/Ensembl gene model. We filtered out all genes having <1 cpm in less than n_minutes samples and Perc MM reads >20% simultaneously. Differential expression analysis was performed using edgeR (59), a statistical package based on generalized linear models, suitable for multifactorial experiments. The threshold for statistical significance chosen was False Discovery Rate (FDR) < 0.05. Gene Ontology (GOEA) and Functional Annotation Clustering analyses were performed by using DAVID Bioinformatic Resources (60, 61), restricting the output to Biological Process terms (BP_FAT). The threshold for statistical significance of GOEA was FDR<0.1 and Enrichment Score z1.5.

### Sequencing PCR products

A fragment of 200 bp was amplified by PCR from genomic DNA extracted from injected mice using the primers listed in Table S12. The PCR products were cloned into a TOPO-TA vector (Invitrogen), and 10 clones for each sample were analyzed by Sanger sequencing.

### Protein immunoprecipitation and mass spectrometry

All experiments were performed in a labeling-free setting and samples prepared using the in StageTip (iST) method. Proteins were precipitated in acetone and then reduced and alkylated in a solution of 6M Guanidine-HCl, 5 mM TCEP, and 20 mM chloroacetamide. Peptides were obtained digesting proteins with LysC (WAKO) for 3 h at 37 °C and with the endopeptidase sequencing-grade Trypsin (Promega) overnight at 37 °C. Collected peptide mixtures were concentrated and desalted using the Stop-and-Go Extraction (STAGE) technique (62). Instruments for LC MS/MS analysis consisted of a NanoLC 1200 coupled *via* a nano-electrospray ionization source to the quadrupole-based Q Exactive HF benchtop mass spectrometer. Peptide separation was carried out according to their hydrophobicity on a home-made chromatographic column, 75 μm ID, 8 Um tip, 300 mm bed packed with Reprosil-PUR, C18-AQ, 1.9 μm particle size, 120 Å pore size, using a binary buffer system consisting of solution A: 0.1% formic acid and B: 80% acetonitrile, 0.1% formic acid. Runs of 75 min after loading were used for the proteome. In both cases, a constant flow rate of 300 nl/min was used. MS data were acquired using a data-dependent top-20, for the proteome, or top-15, for interactome, method with maximum injection time of 20 ms, a scan range of 300–1650Th, an AGC target of 3e6 and a resolution of 120,000. Resolution, for MS/MS spectra, was set to 15,000 at 200 m/z, for the proteome, and 45,000 at 200 m/z, for interactome. AGC target was set 1E5, max injection time to 20 ms and the isolation window to 1.4 Th. The intensity threshold was set at 2.0 E4 and Dynamic exclusion at 30 s.

Raw mass spectrometry data were processed with MaxQuant (1.6.2.10) (63) using default settings (FDR=0.01, oxidized methionine (M) and acetylation (protein N-term) as variable modifications, and carbamidomethyl (C) as fixed modification). For protein assignment, spectra were correlated with the Uniprot Mus Musculus (v.2019), including list of common contaminants. Label-free quantitation (LFQ) and “Match between runs” were enabled. Bioinformatics analysis was performed with Perseus 1.6.2.3 (64). The LFQ intensities were logarithmized, grouped and filtered for min.valid number (min.3 in at least one group). Missing values have been replaced by random numbers that are drawn from a normal distribution. Proteins with Log2 ratios ≥1 and a *p*-value ≤ 0.05 were considered significantly enriched. To identify significant enriched GO terms, we utilized the 1D enrichment tool in Perseus (65).

### Cell culture and treatments

M and Z Huh7.5 cells (31) were cultured in Dulbecco’s modified Eagle’s medium (DMEM) supplemented with 10% fetal bovine serum (FBS), 100 U/ml penicillin and 100 μg/ml streptomycin in 5% CO2 at 37 °C. Cells were treated with DMSO or K67 25 μM (#HY-111126, Medchem) for 48 h before analysis.

### Human liver samples

Human liver samples were collected and anonymized according to applicable human study approvals from subjects homozygous for the p.Glu342Lys pathogenic variant in the *SERPINA1* gene and age-matched control subjects homozygous for the wild-type allele of *SERPINA1*. The study was conducted in accordance with both the Declarations of Helsinki and Istanbul.

### Statistical analyses

Data are expressed as averages ± standard error as indicated in the figure legends. Statistical significance was computed using the *Student’s* 2 tail *t* test or two-way ANOVA as indicated. A *p*-value <0.05 was considered statistically significant.

### Data visualization

Heatmap and Venn diagram were generated using custom annotation scripts.

## Data availability

The data of RNA-seq generated in this study have been deposited in GEO with accession number GSE182961. The mass spectrometry proteomics data have been deposited to the ProteomeXchange Consortium *via* the PRIDE (66) partner repository with the dataset identifier PXD066413.

## Supporting information

This article contains supporting information.

## Conflict of interest

The authors declare that they have no conflicts of interest with the contents of this article.
