# Supplementary material for: p62/SQSTM1-KEAP1 complex prevents clearance of ubiquitinated Z alpha-1 antitrypsin and aggravates liver proteotoxicity
Source: J Biol Chem. 2026 Aug 7;302(10):113410. doi: 10.1016/j.jbc.2026.113410 (PMC13571921; doi:10.1016/j.jbc.2026.113410)
Supplement: Supplementary file 1 [file mmc8.docx]

**Title: p62/SQSTM1-KEAP1 complex prevents clearance of ubiquitinated Z alpha-1 antitrypsin and aggravates liver proteotoxicity**

**Authors:** Nunzia Pastore, Sergio Attanasio, Francesco Annunziata, Claudia D’Agostino, Veronica Maffia, Rita Colonna, Teresa Giuliano, Rosa Ferriero, Iolanda Boffa, Donna Palmer, Philip Ng, Rossella De Cegli, Massimo D’Agostino, Florian Rosenberger, Pavel Strnad, Joseph E. Chambers, Stefan J. Marciniak, Jeffrey Teckman, Pasquale Piccolo, and Nicola Brunetti-Pierri

**Table of contents**

Supplementary Figures…………………..………………………………………………………..5

Supplementary Tables……………………………………………………………………………..5

**Supplementary Figure 1.** **Accumulation of ubiquitinated proteins in PiZ livers.** Immunoblot analysis for total ubiquitinated proteins in liver lysates from 6-week-old **(A)** and 36-week-old **(B)** WT and PiZ mice (n=3/group). GAPDH served as loading control. The GAPDH blot is the same as that shown in Figure 1B because the proteins were analyzed on the same immunoblot. Each dot represents an individual mouse. Averages ± standard deviation are shown. Student’s *t*-test: **p-value < 0.01; *ns* not significant.

**Supplementary Figure 2.** **Reduced** **SERPINA1 secretion in PiZ mice lacking p62/SQSTM1.** Serum ATZ levels in 36-week-old PiZ and PiZ;*p62*^-/-^ mice (n=10 per group). Each dot represents an individual mouse. Averages ± standard deviation are shown. Student’s *t*-test: *p-value < 0.05.

**Supplementary Figure 3.** **Differential Z-hAAT interactome in PiZ and PiZ;*p62^-/-^* livers.** **(A)** Volcano plot of MS analysis showing differentially enriched Z-hAAT interactors in livers from 36-week-old PiZ versus PiZ;*p62^-/-^* mice. (**B**) KEGG pathways enrichment analysis of the interactors identified in PiZ compared to PiZ;*p62^-/-^* mice.

**Supplementary Figure 4.** **NRF2 pathway in PiZ and PiZ;*p62^-/-^* livers.** Gene Set Enrichment Analysis (GSEA) for the NRF2 pathway in the indicated comparisons.

**Table S8.** *Primers for p62/Sqstm1*.

| **Target sequence** |  | **Sequence (5’🡪 3’)** |  |
| --- | --- | --- | --- |
| p62/Sqstm1 | forward | CTGCATGTCTTCTCCCATGAC |  |
|  | reverse | TAGATACCTAGGTGAGCTCTG |  |
| Positive control | forward | CTTACGGGTCCTTTTCCCAAC |  |
|  | reverse | TCCTCCTTGCCCAGAAGATAG |  |

**Table S9.** *Sequences of the guides targeting p62/Sqstm1 cloned into the HDAd vector.*

| **Target sequence** |  | **Sequence (5’🡪 3’)** |  |
| --- | --- | --- | --- |
| sgp62/Sqstm1 | forward | CACCGTGGAGGGTGCTTCGAATAC |  |
|  | reverse | AAACGTATTCGAAGCACCCTCCAC |  |

**Table S10.** *Primary antibodies used for immunoblots and stainings.*

| **Antigen** | **Species in which the Ab was raised** | **Source** | **Code** |
| --- | --- | --- | --- |
| AAT | Rabbit | Dako | A0012 |
| AAT polymer | Mouse | Hycult biotech | HYC-HM2289 |
| β-actin | Mouse | Invitrogen | AM4302 |
| GAPDH | Mouse | Santa Cruz | sc-32233 |
| p62/SQSTM1 | Rabbit | MBL | PM045 |
| p62/SQSTM1 | Mouse | Abnova | H00008878 |
| P-p62 (S351) | Mouse | MBL | M217-3 |
| Ubiquitin | Rabbit | Cell Signaling | 58395 |
| VCP/p97 | Rabbit | Novus Biological | NBPZ-15162 |
| SEL1L | Rabbit | Abcam | Ab78298 |
| Vinculin | Rabbit | Cell Signaling | 13901 |
| H3 | Rabbit | Cell Signaling | 9715 |
| KEAP1 | Rabbit | Proteintech | 10503-2-AP |
| NQO1 | Rabbit | Abcam | Ab34173 |
| NRF2 | Rabbit | Proteintech | 16396-1-AP |

**Table S11**. *Primers for real time PCR*

| **Gene** | **Species** |  | **Sequence (5’🡪 3’)** |
| --- | --- | --- | --- |
| *p62/Sqstm1* | mouse | forward | TGTGGTGGGAACTCGCTATAA |
|  |  | reverse | CAGCGGCTATGAGAGAAGCTAT |
| *SERPINA1* | human | forward | TGCCCAGGTATTTCATCAGC |
|  |  | reverse | CCGTGAAGGTGCCTATGATG |
| *Beta2 microglobulin* | mouse | forward | TGGTGCTTGTCTCACTGACC |
|  |  | reverse | GTATGTTCGGCTTCCCATTC |

**Table S12**. *Primers for amplification and sequencing of the p62/Sqstm1 gene.*

| **Target sequence** |  | **Sequence (5’🡪 3’)** |  |
| --- | --- | --- | --- |
| p62/Sqstm1 | forward | AAGGCATCACACATTCCCAC |  |
|  | reverse | TGTCCATGGGTTTCTCGGAT |  |
